# Modelling biological age based on plasma peptides in Han Chinese adults

**DOI:** 10.18632/aging.103286

**Published:** 2020-06-05

**Authors:** Weijie Cao, Deqiang Zheng, Guohua Wang, Jie Zhang, Siqi Ge, Manjot Singh, Hao Wang, Manshu Song, Dong Li, Wei Wang, Xizhu Xu, Youxin Wang

**Affiliations:** 1 Department of Epidemiology and Health Statistics, School of Public Health, Beijing Key Laboratory of Clinical Epidemiology, Capital Medical University, Beijing 100069, China; 2 The Second Affiliated Hospital of Shandong First Medical University, Tai’an 271000, China; 3 Beijing Neurosurgical Institute, Beijing 100070, China; 4 School of Medical and Health Sciences, Edith Cowan University, Perth 6027, Australia; 5 School of Public Health, Shandong First Medical University and Academy of Medical Sciences of Shandong Province, Tai’an 271016, China

**Keywords:** ageing, multiple linear regression, biological age, plasma peptide, primary prevention

## Abstract

Age-related disease burdens increased over time, and whether plasma peptides can be used to accurately predict age in order to explain the variation in biological indicators remains inadequately understood. Here we first developed a biological age model based on plasma peptides in 1890 Chinese Han adults. Based on mass spectrometry, 84 peptides were detected with masses in the range of 0.6-10.0 kDa, and 13 of these peptides were identified as known amino acid sequences. Five of these thirteen plasma peptides, including fragments of apolipoprotein A-I (m/z 2883.99), fibrinogen alpha chain (m/z 3060.13), complement C3 (m/z 2190.59), complement C4-A (m/z 1898.21), and breast cancer type 2 susceptibility protein (m/z 1607.84) were finally included in the final model by performing a multivariate linear regression with stepwise selection. This biological age model accounted for 72.3% of the variation in chronological age. Furthermore, the linear correlation between the actual age and biological age was 0.851 (95% confidence interval: 0.836-0.864) and 0.842 (95% confidence interval: 0.810-0.869) in the training and validation sets, respectively. The biological age based on plasma peptides has potential positive effects on primary prevention, and its biological meaning warrants further investigation.

## INTRODUCTION

The trends toward an increased aged population (the proportion of individuals aged 65 years and over) is a major public health problem, especially in China [1, 2]. Worldwide, the estimated number of elderly population was 962 million in 2017, and the growing rate was approximately 3% per year [3]. China has the largest elderly population in the world, with more than 225 million elderly people. The number of elderly people in China is projected to be 400 million by 2030 [4]. Ageing is a complex process characterized by progressive degradation of structural and functional integrity, during which the ability to maintain homeostasis is gradually lost, leading to the risk of impaired function and disease susceptibility [5–8]. Furthermore, ageing is a major risk factor for various chronic diseases [8]. However, there is great heterogeneity in health outcomes among elderly individuals of the same age group, suggesting that actual age is not an optimal indicator of the ageing progress [9].

Actual age correlates with the accumulation of biological changes, and individuals with the same actual age undergo these changes at different rates [10]. Biological age is used to measure damage accumulation with age at an individual level and can be quantified from the known biomarkers of ageing [11, 12]. To date, the ageing biomarkers include telomere length [13], DNA methylation [14–16], transcriptomic predictor [17, 18], plasma peptide [19], IgG N-glycosylation [20, 21], facial morphology [22], waist circumference density index [23], among others. Biological age determined by age-related DNA methylation has proven to be better than chronological age, as a predictor of 3-month outcomes after ischaemic stroke [24]. Biological age can issue a timely warning for health care and make people realize that his health is slipping away [12]. Therefore, the regular monitoring of the discrepancy between biological age and chronological age has potential positive impacts on the primary prevention and disease burden.

Biological ageing is associated with reduced reparative and regenerative potential of the body [25]. The ideal candidates to be studied for the purpose of predicting biological age must be representative of the level of homeostatic balance in the body. Plasma peptides, such as hormones, cytokines and growth factors, promote homeostasis in many biological processes [26]. Additionally, some plasma peptides have been found to be associated with age-related diseases, including Alzheimer's disease, hypertension, type 2 diabetes, and colorectal cancer [27–31]. Furthermore, our previous study shows that some peptides are highly correlated with chronological age in a Chinese population, such as fragment of apolipoprotein A-I, fibrinogen alpha, albumin and so on [19]. In particular, the levels of apolipoprotein A-I and fibrinogen alpha fragment gradually increased between 18 and 50 years of age, while albumin significantly degraded in middle-aged individuals. In the present study, we focused on building a biological age model with a set of specific plasma peptides from a Han Chinese population.

## RESULTS

### Description of the subjects

This cross-sectional study included 1890 participants of Han Chinese descent. The summary of demographic variables was shown in Table 1. The median age was 34 years (P_25_ 27 years, P_75_ 45 years) in all subjects, 34 years (27 to 45 years) in male subjects, and 36 years (26 to 46 years) in female subjects (Table 1). All anthropometric variables, except for age and age group variables, were significantly different between male and female subjects (*P* < 0.001). Compared with female participants, the male subjects had greater height, weight, systolic blood pressure (SBP), diastolic blood pressure (DBP), and body mass index (BMI).

**Table 1:** Characteristics of the participants.

**Parameters**	**Total (n=1890)**	**Males (n=1136)**	**Females (n=754)**	***P***
Age, year	34 (27-45)	34 (27-45)	36 (26-46)	0.512
≤ 39	1177 (62.28%)	721 (63.47%)	456 (60.48%)	
40-59	593 (31.38%)	323 (28.43%)	270 (35.81%)	
≥ 60	120 (6.35%)	92 (8.10%)	28 (3.71%)	
Age group, %				0.148
≤ 40 years old	1225 (63.81%)	751 (66.11%)	474 (62.86%)	
> 40 years old	665 (35.19%)	385 (33.89%)	280 (37.14%)	
Height, cm	169 (163-173)	172 (169-176)	162 (158-166)	< 0.001^*^
Weight, kg	67 (58-76)	73 (66-81)	57 (52-63)	< 0.001^*^
BMI, kg/m^2^	23.7 (21.2-25.9)	24.6 (22.5-26.8)	21.8 (19.9-24.1)	< 0.001^*^
SBP, mmHg	120 (110-130)	120 (112-130)	114 (104-124)	< 0.001^*^
DBP, mmHg	78 (70-82)	80 (70-86)	72 (66-80)	< 0.001^*^

*P < 0.05 indicating that the peptide was taken as a significant one. BMI: Body mass index; SBP: Systolic blood pressure; DBP: Diastolic blood pressure.

### Model for predicted biological age

Among 84 detected peptides with masses in the range of 0.6-10.0 kDa, 13 identified peptides with amino acid sequences were used for subsequent analysis (Table 2). In particular, 11 peptides were selected for further analysis based on univariate linear regression (Table 3), except for fragment of complement C3 (m/z 1120.39) and complement C4-A (m/z 1052.53). As shown in Table 4, five plasma peptides, including fragments of apolipoprotein A-I (m/z 2883.99), fibrinogen alpha chain (m/z 3060.13), complement C3 (m/z 2190.59), complement C4-A (m/z 1898.21), and breast cancer type 2 susceptibility protein (m/z 1607.84), were identified by stepwise selection in a multivariate linear regression based on these 11 peptides and all demographic traits (BMI, SBP, DBP, age group). Finally, a biological age model was built based on the five identified plasma peptides and three demographic variables (Table 4). The estimated biological age can be calculated using the following equation (1).

**Table 2:** Characteristics of identified plasma peptide in the participants.

**Peak**	**Amino acid sequence**	**Peak identity**	**Total (n=1890)**
m/z 2044.75	K.VFDEFKPLVEEPQNLIK.Q	Albumin	105.3 (40.0-160.1)
m/z 2065.31	D.APRIKKIVQKKLAGDESAD.-	Pro-Platelet basic protein	35.3 (14.2-75.1)
m/z 2487.01	S.NSRDDGNSVFPAKASATGAGPAAAEK.R	Hyperpolarization-activated cyclic nucleotide-gated potassium channel 1	26.1 (16.7-42.1)
m/z 3428.10	K.YWSQQIEESTTVVTTQSAEVGAAETTLTELR.R	Keratin 18	60.4 (24.3-113.5)
m/z 2883.99	L.LPVLESFKVSFLSALEEYTKKLNTQ.-	Apolipoprotein A-I	36.4 (19.5-59.2)
m/z 1076.14	E.GDFLAEGGGVR.G	Fibrinogen alpha chain	30.2 (20.6-41.8)
m/z 3060.13	K.SSSYSKQ(+.98)FTSSTSYNRGDSTFESKSYK.M	Fibrinogen alpha chain	73.6 (33.3-135.6)
m/z 1120.39	T.HRIHWESAS.L	Complement C3	18.5 (13.1-25.7)
m/z 2190.59	G.SPMYSIITPNILRLESEET.M	Complement C3	45.7 (26.2-80.4)
m/z 1052.53	K.SHALQLNNR.Q	Complement C4-A	20.6 (13.7-29.9)
m/z 1898.21	S.STGRNGFKSHALQLNNR.Q	Complement C4-A	24.3 (14.8-56.8)
m/z 1607.84	P.KC(+57.02)KEMQNSLN(+.98)NDK.N	Breast cancer type 2 susceptibility protein	19.0 (9.97-38.8)
m/z 2211.86	V.YRLPPLRKGEVLPLPEAN(+.98)F.P	Histidine-rich glycoprotein	38.1 (20.0-80.8)

Data were presented as median together with interquartile range. Peptide content in human plasma is measured in intensity.

**Table 3:** Univariate linear regression analysis for each identified peptide.

**Variables**	**Coefficient**	**SE**	***P***	**95% CI**
Albumin (m/z 2044.75)	-0.005	0.002	0.012^*^	(-0.008, -0.001)
Pro-Platelet basic protein (m/z 2065.31)	-0.008	0.005	0.100^*^	(-0.018, 0.002)
Hyperpolarization-activated cyclic nucleotide-gated potassium channel 1 (m/z 2487.01)	0.040	0.014	0.004^*^	(0.013, 0.067)
Keratin 18 (m/z 3428.10)	0.014	0.005	0.002^*^	(0.005, 0.023)
Apolipoprotein A-I (m/z 2883.99)	0.040	0.009	< 0.001^*^	(0.023, 0.058)
Fibrinogen alpha chain (m/z 1076.14)	-0.064	0.017	< 0.001^*^	(-0.097, -0.030)
Fibrinogen alpha chain (m/z 3060.13)	0.009	0.003	0.002^*^	(0.003, 0.015)
Complement C3 (m/z 1120.39)	-0.048	0.029	0.100	(-0.105, 0.009)
Complement C3 (m/z 2190.59)	0.011	0.005	0.034^*^	(0.001, 0.022)
Complement C4-A (m/z 1052.53)	0.002	0.023	0.921	(-0.042, 0.046)
Complement C4-A (m/z 1898.21)	-0.007	0.004	0.074^*^	(-0.014, 0.001)
Breast cancer type 2 susceptibility protein (m/z 1607.84)	0.015	0.009	0.072^*^	(-0.001, 0.032)
Histidine-rich glycoprotein (m/z 2211.86)	0.007	0.004	0.090^*^	(-0.001, 0.016)

*P < 0.10 indicating that the peptide was taken as a significant one. The P value of pro-platelet basic protein (m/z 2065.31) was 0.0996 which rounded to three decimal places (0.100). Complement C3 (m/z 1120.39) and Complement C4-A (m/z 1052.53) were not selected for further multivariate linear regression analysis. SE: Standard error; 95% CI: 95% Confidence interval.

**Table 4:** Multiple linear regression analysis for biological age.

**Variables**	**Coefficient**	**SE**	***P***	**95% CI**
Constant	13.3	1.73	< 0.001^*^	(9.9, 16.7)
BMI	0.122	0.054	0.025^*^	(0.016, 0.229)
SBP	0.107	0.015	< 0.001^*^	(0.079, 0.136)
Age group	21.0	0.387	< 0.001^*^	(20.2, 21.7)
Apolipoprotein A-I (m/z 2883.99)	-0.014	0.006	0.035^*^	(-0.026, -0.001)
Fibrinogen alpha chain (m/z 3060.13)	0.004	0.002	0.050^*^	(3.69 × 10^-6^, 7.50 × 10^-3^)
Complement C3 (m/z 2190.59)	0.011	0.004	0.003^*^	(0.004, 0.019)
Complement C4-A (m/z 1898.21)	-0.005	0.003	0.053	(-1.02 × 10^-2^, 5.50 × 10^-5^)
Breast cancer type 2 susceptibility protein (m/z 1607.84)	0.019	0.006	0.002^*^	(0.007, 0.031)

*P < 0.05 indicating that the peptide was taken as a significant one. The P value of fibrinogen alpha chain (m/z 3060.13) was 0.0498 which rounded to three decimal places (0.050). Adjusted R^2^ = 0.723.

BMI: Body mass index; SBP: Systolic blood pressure; SE: Standard error; 95% CI: 95% Confidence interval.

Biological age = 13.3 + 0.122 × BMI + 0.107 × SBP + 21.0 × age group + (-0.014) × Apolipoprotein A-I fragment (m/z 2883.99) + 0.004 × Fibrinogen alpha chain fragment (m/z 3060.13) + 0.011 × Complement C3 fragment (m/z 2190.59) + (-0.005) × Complement C4-A fragment (m/z 1898.21) + 0.019 × Breast cancer type 2 susceptibility protein fragment (m/z 1607.84)(1)

**Figure 1:**
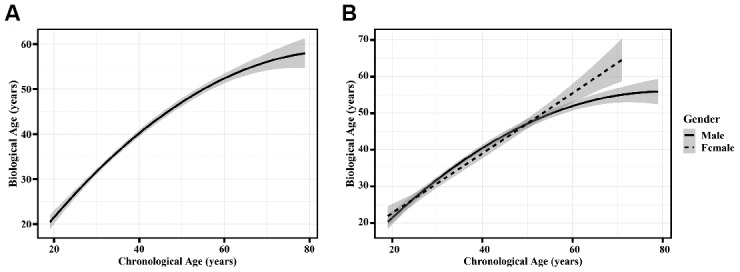
**The correlation between chronological age and biological age.** (**A**) The model performance based on the validation set. (**B**) The model performance presented by sex in the validation set. Dotted and solid curves were fitted to describe correlations between biological age and chronological age in females and males, respectively. The shade region was a pointwise 95% confidence interval.

All samples (from 1890 Chinese Han adults) were randomly divided into the training set (1500 samples) and validation set (390 samples). This model accounted for 72.3% of the variation in chronological age, with a correlation between the actual age and biological age of 0.851 (95% confidence interval (95% CI): 0.836-0.864) in the training set. Furthermore, in the validation set, the biological age was linearly correlated with the actual age (correlation coefficient (r) = 0.842, 95% CI: 0.810-0.869), and the normalized mean square error (NMSE) was 0.30. Visual analysis of the correlations between biological age and chronological age is presented in Figure 1. The predictive effect of the model is considered outstanding when the correlation curve is a straight line and its slope is equal to 1. The 95% CI of the fitted curve broadened with age, suggesting that the variation in biological age and heterogeneity among different individuals increased with actual age. Thus, plasma peptides can serve as potential biomarkers for predicting biological age, and their practical application warrants further research.

## DISCUSSION

Biological age is a health indicator associated with chronological age, senescence and disease, and it can reflect dynamic and alterable health status better than chronological age [32]. In the present study, we built a biological age model correlated with actual age (*r* = 0.842, 95% CI: 0.810-0.869) in the validation set that explained 72.3% of the variation in chronological age, but its predictive ability still needs further verification. To the best of our knowledge, this study is the first attempt to build a biological age model based on plasma peptides in Han Chinese adults.

Matrix-assisted laser desorption/ionization time-of-flight mass spectrometry (MALDI-TOF-MS) is a key tool for peptide analysis of human body fluids, such as plasma, saliva and urine samples [26]. Based on this method, thirteen peptides were identified their amino acid sequences, and five of these peptides were used to construct a biological age model (fragment of apolipoprotein A-I (m/z 2883.99), fibrinogen alpha chain (m/z 3060.13), complement C3 (m/z 2190.59), complement C4-A (m/z 1898.21), and breast cancer type 2 susceptibility protein (m/z 1607.84)). Compared to our previous study, our study found four novel peptides associated with age [19]. The fragment of apolipoprotein A-I (m/z 2883.99) was found to be a biomarker of biological age in our previous study [19], which is consistent with our results. The level of plasma apolipoprotein A-I is associated with premature coronary artery disease [33] and clinical progression of Alzheimer's disease [34]. We previously found fibrinogen alpha chain (m/z 1076.14) fragment is related to ageing [19], whereas the other fragment of ageing-related fibrinogen alpha chain (m/z 3060.13) was used to build this biological age model in our study. Fibrinogen play an positive role in promoting blood haemostasis and leukocyte function regulation in inflammation [35]. However, elucidating the effect of the level of fibrinogen alpha chain fragment (m/z 3060.13) on ageing still requires further research. Complement C3 activates immune function through complement activation [36]. Furthermore, we also found that other peptides (fragment of complement C4-A (m/z 1898.21) and breast cancer type 2 susceptibility protein (m/z 1607.84)) can be used in predicting biological age, whereas their specific mechanisms in ageing remain to be elucidated.

According to the findings of previous studies, this candidate biological age model had a better age correlation in the validation set (*r* = 0.842, 95% CI: 0.810-0.869) than telomere length (*r* = 0.695 (95% CI: 0.575-0.0.815), without validation) [13], transcriptomic predictor (*r* ranged from 0.348 to 0.744 in different independent cohorts) [17] and IgG Fc N-glycosylation (*r* = 0.59 for the Chinese population, and *r* = 0.84 for the European population) [20, 21], but a weaker correlation with DNA methylation age (*r* = 0.96) in their corresponding validation cohorts covering the entire adult life span and different ethnic populations [15]. Compared to the abovementioned micro biological age, the two macro biological ages also showed strong correlations with the actual age (*r* ranged from 0.85 to 0.86 for the three-dimensional facial image-based age predictor and *r* = 0.992 for the waist circumference density index) [22, 23]. Therefore, composite biomarker predictors may have potential for biological age assessment. In addition, our results were consistent with the large heterogeneity in health state of elderly individuals for the variation in biological age increased with chronological age (95% CI widen with age) [9]. We defined a "age group" variable, a binary variable grouped by 40 years old, based on complex changes of different peptides in different age groups (five age groups 18-29, 30-39, 40-49, 50-59, ≥ 60 years) [19]. The "age group" variable defined 40 years old as the demarcation point artificially based on the balance between these groups. Moreover, women older than 40 years of age will experience the transitional stage characterized by a transition from the reproductive to the non-reproductive stage [37]. Epidemiological studies showed a high prevalence of obesity [38], diabetes mellitus [39], and stroke [40] in adults older than 40 years.

There are several limitations of our study that should be acknowledged. First, due to the limitations of experimental conditions, there may be bias in the peptide analysis because we did not control for pH, removal of oxygen, storage under argon and enzyme inhibitors of all plasma samples, though we controlled for storage time and temperature [41]. Second, although MS-based peptide analysis has been widely used, the detection and identification process is complicated and time-consuming, and the pre-treatment of plasma has a great influence on peptide analysis [42]. Therefore, the pre-treatment of plasma samples and peptide analysis still requires methodological advancement. Finally, people aged 60 years or older comprised a relatively low proportion of the population (6.35%), which may lead to selection and information bias (Table 1). This biological age model needs to be explored in larger and more representative samples, including those of a non-Asian ethnicity, as our study only included Chinese Han adults.

Our finding has certain implications for ageing. This study is the first attempt to develop a biological age model based on plasma peptides in Han Chinese adults. Biological age based on plasma peptides may have the potential to indicate homeostasis abnormalities and the rate of ageing. Our study provided evidence for further research in peptide-based biological age. This evidence may help us to understand the underlying mechanisms of ageing through five age-related peptides.

In conclusion, our study suggested that plasma peptide profiles can be used to build a biological age model. This candidate model involving peptides and clinical traits was able to account for 72.3% of the variation in actual age, and this biological age correlated with chronological age (*r* = 0.842, 95% CI: 0.810-0.869) in the validation set. However, the practical applications of this model in primary prevention warrant further investigation.

## MATERIALS AND METHODS

### Subjects

This cross-sectional study recruited 1927 participants of Han Chinese ancestry during regular health check-ups at Xuanwu Hospital, Capital Medical University, Beijing, China. Individuals who were 18 years old or older were eligible. In addition, subjects with a history of somatic or psychiatric abnormalities in their medical records and those who had used medication two weeks prior to the study were excluded. Subjects who had a history of cerebral infarction, cerebral haemorrhage, other cerebrovascular diseases, congenital heart disease, acute myocardial infarction, liver disease, renal failure, malignant tumour, chronic obstructive pulmonary disease, or rheumatoid arthritis were also excluded. In this study, 37 participants who had missing data for one or more clinical traits were subsequently excluded. Finally, a total of 1890 participants were included in the subsequent analysis. Further details of the study design, recruitment procedure, and physical examination were previously described [19].

### Ethics approval

Written informed consent was obtained from each participant, and all procedures were implemented in accordance with the regulations of the ethics committee of Capital Medical University, Beijing, China.

### Collection of plasma samples

The plasma samples for peptide analysis were collected according to a standard protocol. Fresh fasting blood samples were collected from the cubital vein into blood collection tubes (containing ethylenediaminetetraacetic acid). The plasma was separated by centrifugation at 3,000 rpm for 15 min and then stored at −80 °C until peptide analysis. The number of freeze-thaw cycles of all samples is basically the same during this process. After the plasma samples of all the participants were collected, peptide analysis was completed at the shortest possible time.

### Magnetic bead-based sample preparation for peptide analysis

As in previous studies [27, 28, 43], all plasma samples were fractionated using weak cation exchange magnetic beads to gather and enrich the proteins or peptides, according to the instructions provided by the supplier (ClinProt™, Bruker Daltonics, Billerica, USA) [44, 45]. The samples were purified and isolated through three steps: binding, washing, and elution. The specific details of this process were published in a previous study [19]. Then, the resulting eluates were stored in a –20 °C freezer until further MS analysis.

### Peptides profiling and processing of spectral data

Peptide profiling was performed by MALDI-TOF-MS [28, 43]. First, the eluted samples were diluted in a matrix solution of α-cyano-4-hydroxycinnamic acid and ethanol and acetone, which was prepared daily. Then, 1 μl of the diluted samples was pipetted onto a MALDI-TOF-MS target (AnchorChip™, Bruker Daltonics, Billerica, USA) and dried at room temperature before analysis. Finally, MALDI-TOF-MS measurements were performed using the Autoflex TOF instrument (Bruker Daltonics, Billerica, USA). Profile spectra were acquired from an average of 400 laser shots per sample, with the defined mass range of peak intensities (measured as m/z) of 600–10,000 Da.

Quality control was carried on before the MS analysis, with 11 peptides as external standards where the average molecular weight deviation was no more than 100 μg/g. After testing every 8 samples, each standard preparation was re-calibrated. Additionally, 13 reference samples were run as external standards. The system performance is considered acceptable when the coefficient of variability is less than 30%. All reference peptides and samples were prepared in the same matrix solution as above. All of the solutions and buffers were prepared using MS-grade reagents.

The MALDI mass spectra of peptides were analysed using ClinProTools (ClinProt software version 2.0, Bruker Daltonics, Billerica, USA) to subtract the baseline, normalize the spectra (using total ion current), and determine the peak m/z values and intensities in the mass range of 600–10,000 Da. In brief, local noise estimates were applied to estimate the background, then the background was subtracted from each spectrum. Peptide peaks with a signal-to-noise ratio higher than 5.0 were detected and defined. The cut-off value of the signal-to-noise ratio was set at 5.0 because this value was a good compromise between over detection and sensitivity. A mass shift of no more than 0.1% was determined for the spectra alignment. The peak area was used for quantitative standardization. To determine the peak m/z values or intensities in the target mass range, a ± 2 Da mass accuracy for each spectrum was tolerated [46]. To evaluate the experimental reproducibility, triplicate measurements were performed to examine the standard deviation on the same MALDI-TOF-MS instrument. In our study, the standard deviation was less than 10%, so the reproducibility for the MALDI-TOF MS instrument was considered acceptable.

### Identification of the amino acid sequences of the peptides

The amino acid sequences of the peptides were identified using the nanoliquid chromatography–electrospray ionization–tandem mass spectrometry (nano-LC/ESI–MS/MS) system, which is comprised of an Aquity UPLC system (Waters, Milford, MA, USA) and an LTQ Obitrap XL mass spectrometer (Thermo Fisher Scientific, Bremen, Germany) equipped with a nano-ESI source. In brief, the peptide solution was loaded onto a symmetry C18 trap column (nanoACQUITY) (180 μm × 20 mm × 5 μm) and then analysed by symmetry C18 analytical column (nanoACQUITY, Waters, Milford, MA, USA) (75 μm × 150 mm × 3.5 μm). The mobile phases A, mobile phases B, flow rate and gradient elution were operated according to the published paper [19]. The running mode of the MS instrument was operated in a data-dependent model. The range of the full scan was 400–2,000 m/z with a mass resolution of 100,000 at m/z 400. The FDR cut-off value was set to 0.01 during the whole identification process. The eight strongest monoisotopic ions were the precursors for collision-induced dissociation. The MS/MS spectra were restricted to two consecutive scans per precursor ion followed by a 60-sec of dynamic exclusion.

**Figure 2:**
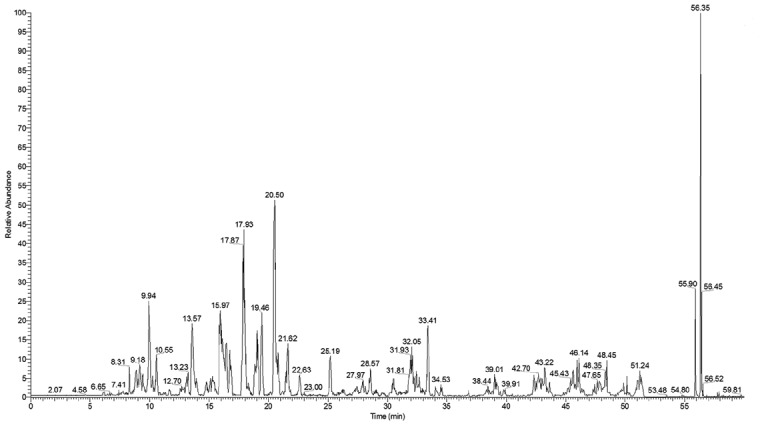
**The total ion current chromatograms of secondary ion mass spectrometry.**

To identify the peptides, the chromatograms were analysed using BioWorksBrowser^TM^ 3.3.1 SP1 software (Thermo Fisher Scientific, Bremen, Germany). The resulting mass lists were located on the Sequest™ (IPI Human v3.45) database (Thermo Scientific, Waltham, MA, USA). Due to the generation of the peak list, the parent ion and fragment mass relative accuracy were set at 50 μg/g and 1 Da, respectively. MS/MS product-ion mass spectra were presented in Figure 2.

### Measurements

The dataset consisted of 7 main demographic variables: age (years), gender, height (cm), weight (kg), BMI (kg/m^2^), SBP (mmHg), DBP (mmHg). Considering that participants at different ages might have large variation in physiological functions [37–40], the constructed model included a “age group” variable defined by a binary indicator, where people aged 40 years and below were represented as 1, and people aged above 40 years were represented as 2. In addition, 84 peptides were detected, with masses in the range of 0.6-10.0 kDa. Among these peptides, 13 peptides were successfully identified as known amino acid sequences. These demographic variables above and the 13 identified peptides were used for subsequent analysis.

### Statistical analysis

Continuous variables were expressed as median and interquartile ranges. Frequencies and percentages were used to express the categorical variables. Continuous variables in the two gender groups were compared using the Mann-Whitney *U* test. The *χ^2^* test was used to compare proportions for categorical variables. Multivariate linear regression was used for the biological age model. The samples were randomly divided into the training set (1500 samples) and validation set (390 samples). The training set and the independent validation set were used for modelling and model validation, respectively. First, a univariate linear regression model was implemented for preliminary selection. If the peptide had a *P* value lower than the entering threshold (*P* < 0.10), then the peptide could be used for further variable screening. Second, all candidate peptides were entered in a multivariate linear regression with stepwise selection adjusting for all demographic variables. The direction argument and entering threshold of stepwise regression were set to “both” and “0.10”, respectively. The criteria for variable selection were based on the Akaike information criterion. Finally, variables identified by stepwise selection were used to build the final biological age mode. The performance of this biological age model was evaluated by the coefficient of determination (R^2^) and NMSE of prediction errors in the independent validation set. Except for variable screening of peptides in regression analysis, a two-tailed *P-*value < 0.05 was considered statistically significant. All statistical analyses were performed using R version 3.3.3 (R Foundation for Statistical Computing, Vienna, Austria).
